# Lower Noise Annoyance Associated with GIS-Derived Greenspace: Pathways through Perceived Greenspace and Residential Noise

**DOI:** 10.3390/ijerph15071533

**Published:** 2018-07-19

**Authors:** Angel M. Dzhambov, Iana Markevych, Boris Tilov, Zlatoslav Arabadzhiev, Drozdstoj Stoyanov, Penka Gatseva, Donka D. Dimitrova

**Affiliations:** 1 Department of Hygiene and Ecomedicine, Faculty of Public Health, Medical University of Plovdiv, 4002 Plovdiv, Bulgaria; gatseva_p@mail.bg; 2 Institute and Clinic for Occupational, Social and Environmental Medicine, University Hospital, LMU Munich, 80336 Munich, Germany; iana.markevych@helmholtz-muenchen.de; 3 Institute of Epidemiology, Helmholtz Zentrum München—German Research Center for Environmental Health, 85764 Neuherberg, Germany; 4 Medical College, Medical University of Plovdiv, 4000 Plovdiv, Bulgaria; btilov@abv.bg; 5 Department of Psychiatry and Medical Psychology, Faculty of Medicine, Medical University of Plovdiv, 4002 Plovdiv, Bulgaria; zlatolini@gmail.com (Z.A.); stojanovpisevski@gmail.com (D.S.); 6 Department of Health Management and Healthcare Economics, Faculty of Public Health, Medical University of Plovdiv, 4002 Plovdiv, Bulgaria; donka_d@hotmail.com

**Keywords:** green space, greenness, noise exposure, noise perception, soundscape

## Abstract

Growing amounts of evidence support an association between self-reported greenspace near the home and lower noise annoyance; however, objectively defined greenspace has rarely been considered. In the present study, we tested the association between objective measures of greenspace and noise annoyance, with a focus on underpinning pathways through noise level and perceived greenspace. We sampled 720 students aged 18 to 35 years from the city of Plovdiv, Bulgaria. Objective greenspace was defined by several Geographic Information System (GIS)-derived metrics: Normalized Difference Vegetation Index (NDVI), tree cover density, percentage of green space in circular buffers of 100, 300 and 500 m, and the Euclidean distance to the nearest structured green space. Perceived greenspace was defined by the mean of responses to five items asking about its quantity, accessibility, visibility, usage, and quality. We assessed noise annoyance due to transportation and other neighborhood noise sources and daytime noise level (L_day_) at the residence. Tests of the parallel mediation models showed that higher NDVI and percentage of green space in all buffers were associated with lower noise annoyance, whereas for higher tree cover this association was observed only in the 100 m buffer zone. In addition, the effects of NDVI and percentage of green space were mediated by higher perceived greenspace and lower L_day_. In the case of tree cover, only perceived greenspace was a mediator. Our findings suggest that the potential for greenspace to reduce noise annoyance extends beyond noise abatement. Applying a combination of GIS-derived and perceptual measures should enable researchers to better tap individuals’ experience of residential greenspace and noise.

## 1. Introduction

Residential noise is a ubiquitous environmental stressor that has been linked to a wide range of non-auditory health outcomes, including cardiometabolic diseases, adverse pregnancy outcomes, and mental ill-health, to name a few [1]. Reduction of noise annoyance, which serves as a proxy for noise exposure and is itself detrimental to health, is one of the possible explanations why people residing in green neighborhoods have better health compared to their counterparts [2]. Greener neighborhoods have less artificial noise-emitting sources [2]; moreover, vegetation can reduce noise levels by physical disruption of sound waves propagated from the source to the receiver [3]. Nevertheless, existing elements in the urban green network, such as street trees, have limited capacity as noise barriers and may even increase pedestrian noise exposure, for instance, in street canyons, where tree canopies can reflect sound waves to the level of pedestrian’s ears [4,5]. Additionally, a large proportion of the variation in residents’ annoyance is caused by non-acoustic factors [1,6]. Therefore, changes in annoyance may be disproportionate to the actual reduction in noise level [1,4]. 

Accumulating evidence is showing that greenspace can reduce traffic-related annoyance via psychological mechanisms, including visual screening of the noise source, increased restorative quality of the residential environment, and masking of unwanted noise with pleasant nature sounds [4]. Additionally, green spaces near the home may strengthen residents’ feeling of control over their acoustic environment by allowing respite from traffic noise, thereby diminishing their noise annoyance [7]. Investigating the latter hypothesis, Riedel et al. observed an indirect effect of near-dwelling greenspace on noise annoyance through perceived noise control [7]. Residing in quiet green areas has been associated with higher health-related quality of life, and that could be due in part to the “quietness” characteristic that people ascribe to greener areas [8]. Even if green spaces are not actively visited, the very knowledge that the residential environment has such natural areas may enhance residential satisfaction and possibly improve noise perception [4,9]. These psychological effects could explain findings that living in a neighborhood with more trees seemed to buffer the negative effect of traffic noise on mental health [10].

Most previous studies on psychological buffering of noise annoyance employed self-reported measures of greenspace and mostly considered only annoyance due to road traffic noise [11]. Van Renterghem and Botteldooren’s study was one of the prominent exceptions, as they examined the effect of both objectively-measured and self-reported green view from home on general and traffic noise annoyance [12]. Another such example is the study by Riedel et al. who used land use maps to assess residential greenspace [7]. Spatial and perceived indices should be used together to better understand the effect of greenspace on health, including noise perception [2]. Metrics derived from Geographic Information Systems (GIS) would be useful when investigating the indirect path linking objective greenspace to noise annoyance through reduced noise levels, whereas self-reports would better highlight residents’ greenspace experience [2]. Another issue that should be considered is that both objective and perceived greenspace can be defined differently, and these metrics may be related differently to noise annoyance [13]. Consistent with this idea, a meta-analysis showed lower odds of high noise annoyance in people who had a green view from their home, whereas the overall greenness (i.e., vegetation degree) in the neighborhood did not reduce noise annoyance [11]. Another study examined the effect of different objective greenspace measures and observed a beneficial effect only for tree cover density in the 100 m buffer zone, but not for overall greenness [14]. 

**Figure 1:**
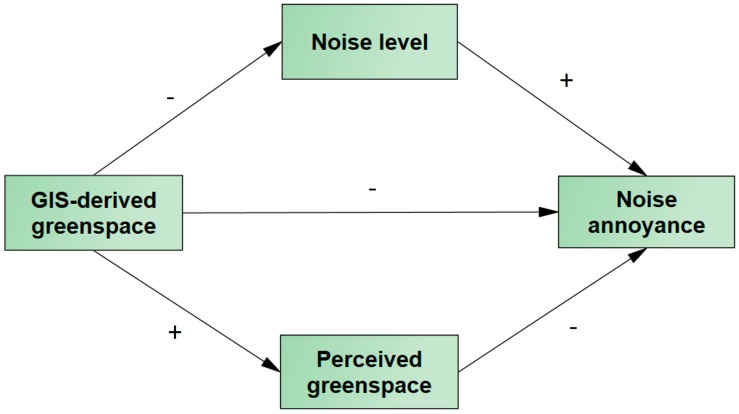
Conceptual diagram showing theoretically-indicated pathways linking Geographic Information System (GIS)-derived greenspace to noise annoyance. (Positive associations are marked with “+”, and negative associations, with “−”.)

To our knowledge, no previous study has investigated whether the effect of greenspace on noise annoyance is mediated by a reduction in noise level and higher perceived greenspace. Such knowledge could help us understand whether relying on objective measures in urban planning and forestry is sufficient to gain insight into these human-environment interactions. In the present study, we examined the association between objectively-measured greenspace and noise annoyance, with a focus on underpinning pathways through residential noise and perceived greenspace in the neighborhood. We tested the model shown in Figure 1 using different measures of greenspace. 

## 2. Materials and Methods

### 2.1. Study Design and Sampling

We used data from a cross-sectional survey conducted between October and November 2017 in students from the Medical University of Plovdiv, Bulgaria. Plovdiv is the second largest city in the country with a population of around 342,000 and a territory of around 102 km^2^. Public green spaces in the city account for 75.3% (381.5 ha) of all green areas, which is 11.2 m^2^ per capita. The distribution of these green spaces is scattered, and in some parts of Plovdiv, there are less than 4 m^2^ of green space per capita [15]. At the same time, the 2016 noise mapping campaign indicated that 77% of residents were exposed to day-evening-night road traffic noise above 60 dB(A) [16].

The aim of our study was to investigate the mental health-supporting effects of residential greenspace. To be included in the study, students had to be aged from 18 to 35 years and reside in Plovdiv or its surroundings for the last six months. We targeted potential participants with different ethnic and cultural backgrounds, ages, and program enrollments to ensure sufficient variation in the data. During a class or lecture, members of the research group advertised the study, informing the students about its general objectives, and asked them to complete a questionnaire. In addition to questions on sociodemographic factors and residential environment, participants were asked to report their current living address for subsequent assignment of geographic variables. The study was conducted in accordance with the Declaration of Helsinki, and the protocol was approved by the Ethics Committee of the Medical University of Plovdiv (1/02.02.2017). All participants provided their informed consent for inclusion before they participated in the study. No incentives were offered. A more detailed account of the survey design has been reported previously [17].

**Figure 2:**
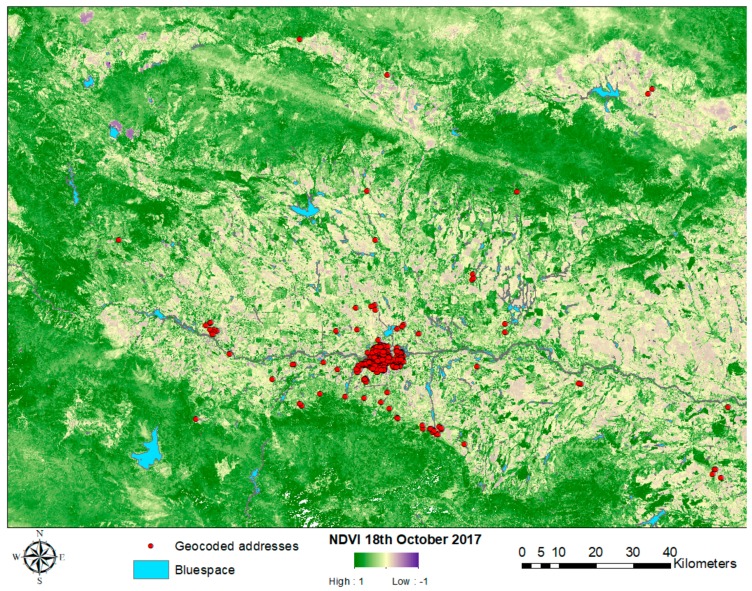
Map of residential addresses superimposed over Normalized Difference Vegetation Index (NDVI) geographic layer.

Of the 1000 students invited, 823 (82%) agreed to participate. Residential addresses were manually converted into geocodes with the help of Google maps. Of the 823 students, 720 provided sufficient data, including residential addresses, to be included in the study. The majority of them (n = 642, 89.2%) lived in the city of Plovdiv (Figure 2). 

### 2.2. Greenspace

Based on previous evidence that different types and properties of greenspace may have different potential to reduce noise annoyance [14], objective greenspace was defined by several GIS-derived metrics: Normalized Difference Vegetation Index (NDVI), percentage of green space, tree cover density, and the Euclidean distance to the nearest structured green space. NDVI [18] served as a measure of surrounding greenness. NDVI is commonly used as a proxy for overall vegetation level and ranges from −1 to +1, with positive values close to 1 indicating dense vegetation [19]. The NDVI equation is based on the difference in surface reflectance in two vegetation-informative wavelengths: visible red and near infrared light. For these calculations, we used six Sentinel 2 MultiSpectral Instrument satellite images with a resolution of 10 × 10 m for the needed bands, obtained on 16 and 18 October 2017. Because bluespace is thought to share the capacity of greenspace to improve noise perception, we wanted to differentiate the effect of greenspace from the effect of bluespace. So, we removed all water pixels from the satellite images prior to assigning NDVI to the geocodes by using the Open Street Maps (OSM) water layer [20]. Tree cover density was calculated based on the Tree Cover Density 2012 map developed by the European Environmental Agency at a resolution of 20 × 20 m. Percentage of structured green space was calculated from the Urban Atlas 2012 land use map. Mean NDVI, mean tree cover density, and percentage of structured green space were abstracted in circular buffers of 100, 300 and 500 m around the residence [17,20]. Euclidean distance to the edge of the nearest structured urban green space was calculated based on the OSM data, and included parks, allotments, and recreation grounds. Geographic data management and calculations were performed using ArcGIS 10.3–10.4 Geographical Information System (GIS) (ESRI, Redlands, CA, USA).

Perceived greenspace was assessed with five items asking respondents about different aspects of their experience of, and interaction with, greenspace in their neighborhood [17]. Specifically, we considered the overall perceived neighborhood greenness, visible greenery from home, accessibility to the nearest structured green space, time spent in green space, and quality of green space. Items could be answered on a 6-point scale. The score for this measure was the mean of item responses, with higher values indicating greater perceived greenspace in the living environment. Cronbach’s alpha for this scale was 0.81.

### 2.3. Noise Level

Noise level was calculated by a combination of measurements and modelling at each participant’s address with the help of a land use regression (LUR) model. The LUR was developed specifically for this study and was based on noise measurements recorded by the Regional Health Inspection at 40 locations in Plovdiv in 2016. Measurements were conducted over a 12-hour period from 7:00 a.m. to 7:00 p.m. (L_day_) according to ISO 1996-2:1987. Predictor variables derived from GIS were considered in the regression equation, following a supervised forward stepwise selection procedure, as described by Aguilera et al. [21]. The final LUR had an adjusted correlation coefficient (R^2^) of 0.72 and leave-one-out cross validation R^2^ of 0.65. More details about model development have been reported previously [17]. 

### 2.4. Noise Annoyance

Noise annoyance was calculated by the mean of responses to two items. Items asked about annoyance/disturbance by traffic noise and other neighborhood noise sources included: “How much does road traffic noise bother, disturb, or annoy you?” and “How much does noise from neighbors/construction/recreational establishments bother, disturb, or annoy you?”. The response scale ranged from “0 = Not at all” to “4 = Extremely” and followed the recommendation by the International Commission on Biological Effects of Noise [22]. The correlation between the two items was strong enough to justify their combination into one scale, r = 0.47 (*p* < 0.001). The mean of the responses served as a measure of noise annoyance in the living environment.

### 2.5. Confounders

We also considered several potential confounding factors, including participants’ age, sex, ethnicity, duration of residence at the address, and average time spent at home per day. Individual-level economic status was assessed by a single item: “Having in mind your monthly income, how easy is it for you to “make ends meet”, meet your expenses without depriving yourself?”. Responses could range from “0 = Very difficult” to “5 = Very easy”. Recently experienced stressful life events were assessed with the item “Have you lately experienced a stressful life event, such as death/illness of a relative, separation from a loved one, or being fired?”. The geocodes were used to assess population density in a 500 m buffer zone around the address from the 2011 Census population grid of Bulgaria and whether participants lived in Plovdiv or any other settlement nearby. We also considered the presence of bluespace in circular buffers of 100 m, 300 m and 500 m, and the Euclidean distance to the edge of bluespace nearest to the residence. For these calculations, we extracted data on all types of water bodies and wetlands from the Urban Atlas 2012. Given seasonal variations in noise annoyance [23], we also considered the month of data collection. 

### 2.6. Statistical Analysis

Most variables in the dataset had less than 5% missing values, except L_day_ (11.3% missing) because the LUR for noise was only applied to address points in Plovdiv, where its validity was confirmed. All missing data were replaced using the expectation maximization algorithm [24,25]. Interrelations between the variables were examined with Pearson correlations. Given the robustness of parametric analyses to deviations from the normal distribution and that Likert scales with five or more categories can be treated as continuous variables [26,27], noise annoyance and perceived greenspace were included in the parametric tests.

For the main analysis, we tested mediation models linking greenspace to noise annoyance. According to mediation theory, the total effect of greenspace is the sum of the direct effect of greenspace on annoyance, controlling for perceived greenspace and L_day_, and the two indirect paths through perceived greenspace and L_day_ working in parallel. We employed the pre-specified Model 4 of PROCESS v. 2.16 [28]. PROCESS is a free macro for SPSS that simplifies the implementation of mediation analysis with observed variables, based on a set of preprogrammed conceptual and statistical models. It uses ordinary least squares regression to estimate the parameters of each of the equations, including all the path coefficients, standard errors, *t*- and *p*-values, and confidence intervals [29]. In our case, the bias-corrected 95% confidence intervals (CIs) of indirect paths were computed using bootstrapping (5000 samples). The model was applied to each of the objective greenspace metrics. All models were adjusted for a priori selected confounders: age, sex, ethnicity (Bulgarian vs. other), economic status, duration of residence (<5 vs. ≥5 years), time spent at home per day (<8 vs. ≥8 h), stressful life events (no vs. yes), population in the 500 m buffer, settlement (Plovdiv vs. other), presence of bluespace in the respective buffer (or distance to bluespace in the model with distance to green space), and month of data collection (October vs. November). Coefficients for NDVI and distance to green space were rescaled to an interquartile range increment, and for tree cover density and percentage of green space, to a 5% increment. In a sensitivity analysis, the model was fitted separately for annoyances due to traffic noise and other neighborhood sources.

Results were considered statistically significant at the *p* < 0.05 level, and mediation was considered present when the indirect path significantly exceeded zero, regardless of the significance of the total effect [30].

## 3. Results

Participants’ age was not normally distributed, with a median of 21.00 years (interquartile range = 3.0). The sample included substantially more women than men (66% vs. 34%), and most were Bulgarian (74%). The majority of the sample was from the city of Plovdiv (89%), lived in their home for less than five years (62%), and spent at least eight hours at home per day (55%). Correlations between the key variables were in line with theory (Table 1). That is, higher noise level at the residence was associated with higher noise annoyance, whereas those living in a greener neighborhood, or perceiving their neighborhood as greener, reported lower annoyance. In addition, higher residential greenspace was related to lower noise level and higher perceived greenspace. 

Tests of the total effect of greenspace showed that higher NDVI and percentage of green space were associated with lower noise annoyance across all buffers (Table 2). For tree cover density, this association was significant only in the 100 m buffer zone. Distance to green space had no effect. Further, only NDVI in the larger buffers (300 m and 500 m) had a direct effect on noise annoyance, and for all other buffers and metrics, the effect was only indirect. More specifically, the associations between NDVI and percentage of green space and noise annoyance were mediated by higher perceived greenspace and lower L_day_, and the coefficients associated with these paths did not differ significantly. Perceived greenspace accounted for around 20–25% of these total effects. In terms of tree cover, only perceived greenspace was a mediator, explaining from 14 to 75% of the total effect depending on the buffer.

A similar overall trend was revealed when we fitted the mediation model separately to traffic and neighborhood noise annoyances. However, associations between greenspace and neighborhood noise annoyance appeared to be stronger and more consistent than for traffic noise annoyance. (Appendix A, Table A1). Conversely, the path through L_day_ was significant only in the model with traffic noise annoyance.

## 4. Discussion

### 4.1. General Discussion

This study examined associations between GIS-derived greenspace and noise annoyance in Bulgarian students. Higher surrounding greenness and percentage of green space in the residential environment were associated with lower noise annoyance across all buffers, whereas greater tree cover density seemed beneficial only in the 100 m buffer zone. Proximity to green space had no effect. The effects of surrounding greenness and percentage of green space were mediated by higher perceived greenspace and lower noise level, and these paths were equally important. In the case of tree cover, only perceived greenspace was a mediator.

Overall, our results are in line with a substantial body of literature on residential greenspace and its capacity to reduce noise annoyance [12,13,31,32,33,34,35]. To the best of our knowledge, only our earlier study in Plovdiv [14] examined this relationship across different definitions of greenspace, as measured by different remotely-sensed vegetation indices. However, in that earlier study, results were diametrically opposite, as NDVI and percentage of green space (in the 100 m and 300 m buffers) were associated with higher road traffic noise annoyance [14]. Although such contrasting findings cannot be fully understood here, we offer an angle to these differences. In the present study, participants were older (18–35 vs. 15–25 years), some were foreigners, and the two items used tackled noise annoyance both at home and in the neighborhood, as opposed to noise annoyance at home only, in our previous study. The difference in definitions of annoyance deserves specific consideration because, depending on the spatial context, different mechanisms could explain the effect of greenspace on annoyance. Conceivably, having a green view from the home windows may be more beneficial for noise perception at home than the overall greenness in the neighborhood [11]. From this perspective, tree cover in the 100 m buffer zone possibly served as a proxy for visible greenery from home [2]. This effect may be attributed to visual screening of the noise source by street trees, stress reduction, or reinforcing the notion that noise levels are actually lowered by street trees [4]. Further, living in a green environment and having dwelling-related green might be associated with lower noise annoyance via increased perceived control over the acoustic environment [11]. That is, a person’s feeling of being unable to escape and retaliate against the noise source might be suppressed by the knowledge that their home has tranquil green spaces in its vicinity [7]. However, NDVI and percentage of green space in larger buffers represent general availability of greenspace in the neighborhood, which may be relevant for noise annoyance experienced outside of the home. Green areas typically serve as places of a high restorative quality where people can find refuge from noise and also engage in social contacts and physical activity [2,36]. In smaller buffers, tree cover is a more precise measure considering only trees, which are observable from afar owing to their height, whereas NDVI can grasp vegetation not visible from home (on roofs, herbaceous vegetation, etc.). Percentage of green space is based on land use data; hence, it can encompass green spaces with varying, sometimes scarce, vegetation levels. As for distance to urban green space, no association was observed here, in contrast with previous reports [31,32] that found that having access to green space within 200–400 m from home is beneficial. Non-linearity in the effect or the urban fabric of Plovdiv may explain such findings. 

Tests of the mediation models showed that only NDVI in the 300 m and 500 m buffers had a direct effect on noise annoyance, and for all other combinations and metrics, the effect was only indirect. That is, higher greenspace is related to lower noise level and higher perceived greenspace, and in turn to lower annoyance. These two mediators worked in parallel and seemed equally important, explaining some 20–25% of the total effect. Our findings that both perceived greenspace and lower noise level were equally important mediators imply that greenspace may support noise perception beyond reduction in noise level. This could have value for the planning and management of urban green systems. Installation of solid noise barriers, albeit associated with a considerable reduction in noise exposure, is one of the least cost-efficient noise abatement approaches [37,38]. Street trees also have limited potential to block sound propagation despite what residents might believe [4]. Conversely, urban green systems could be retrofitted, such as by rejuvenation, installing water features, and attracting songbirds, to improve the acoustic and restorative quality in residential areas at a relatively low cost. Such interventions to enhance residents’ satisfaction with the neighborhood and to reduce their noise annoyance seem more feasible than changing the existing urban fabric, such as by changing traffic-related infrastructure. To understand the personal factors that could affect soundscape appraisal beyond physical features of green spaces, case studies and noise-related social surveys in areas where noise has been identified as an issue could be a useful tool for policy-makers and planners [39]. In any case, improving the acoustic comfort of residents is just one of the many benefits delivered by urban green infrastructure, and this evidence should be heralded to increase the awareness of stakeholders and narrow the gap between science and greenspace policy [40]. 

In the sensitivity analysis, we found a stronger effect of greenspace on neighborhood noise annoyance than on traffic noise annoyance. We think this effect could be due to the fact that, for some residents, other noise sources may be more annoying than road traffic, possibly due to differences in spectral characteristics, residents’ learned helplessness, or other psychoacoustic, social, and cognitive determinates of noise perception [1,41]. We also found that noise level was a mediator only in the traffic noise annoyance model, possibly because the L_day_ variable, for the most part, captured variations in outdoor traffic noise. However, noise originating from within the residential building or from neighbors and recreational activities should be more relevant for neighborhood noise annoyance.

Our findings encourage the consideration of both objective and perceptual measures of greenspace. Self-reports may better account for the actual interaction with greenspace, and therefore reduce misclassification in remote sensing exposure assessments, which is due to some well-known issues with GIS-derived metrics like the NDVI and land use indices (e.g., poor discrimination between types of greenspace and disregarding their actual quality, accessibility, and visibility from pedestrian’s point of view) [2,19]. Some remedies include new indices capturing multiple aspects of greenspace by integrating information from different sources, including remote sensing, local land use databases, private ownership data, and quality appraisals [42]; using GPS technology to track participants’ movement and assess greenspace along their actual travel routes [43]; and calculating visible vegetation from geotagged eye-level panoramic images [44]. However, perceptual measures not only compensate for the lack of precision of vegetation indices and land use-derived green space metrics, but also access the mental representation of residents’ living environment, considering individual differences, such as the attention that people pay to their surroundings, their preference for specific attributes involved in the appraisal of greenspace quality, personal salience, and intentions to engage in particular greenspace-related behaviors (e.g., “green sport”) [45,46]. Given the mismatch between GIS-derived and perceptual measures [46,47] and the sporadic use of more sophisticated exposure assessment techniques, applying a combination of objective and self-reported greenspace measures should enable researchers to better capture individuals’ experience of greenspace and to understand how it may relate to noise annoyance [45,46,48]. 

### 4.2. Strengths and Limitations

As a novel contribution here, we collected data on various objective and perceived greenspace measures and employed state of the art exposure assessment—e.g., NDVI’s resolution was higher than in most greenspace research (10 × 10 m vs. 30 × 30 m) [2], and noise level was assessed by a specifically developed and up-to-date LUR model. Another novel feature of this study is that we assessed noise annoyance due to neighborhood sources other than road traffic. We focused on an understudied age group from South-East Europe. By sampling students from one university, we reduced overdispersion in the data and also controlled for environmental influences (e.g., traffic noise, greenness) on campus. The high response rate (>80%) is also a strength.

However, several limitations should be discussed. First, this study was of cross-sectional design like all earlier studies in the field [11]. This precludes us from drawing causal inferences about revealed associations. Additionally, cross-sectional tests of mediation might entail bias and produce overconfident results [49]. Recall bias cannot be ruled out either. Therefore, our findings should be replicated in longitudinal analyses. It is important to know the length of time a person needs to be “exposed” to greenspace for noise perception to be improved. Also of interest would be describing how the effect of greenspace varies throughout the year depending on seasonal variations in residential vegetation and in different geographic contexts.

Second, the inclusion of foreigners and sampling from a medical school means that our sample was very specific and not representative of the general population of Plovdiv of that age range. Still, this lack of external validity does not have an effect on the internal validity of our study, because we controlled for a wide range of sociodemographic and residential factors.

Third, we likely overestimated noise level at addresses located near minor roads and in smaller settlements, due to the limited observed range of measurements used to construct the LUR. Even though that should have diminished the real association between L_day_ and noise annoyance, we still detected significant mediation through L_day_.

Forth, concerns that single noise annoyance questions do not adequately reflect the whole experience of noise have been expressed [50,51]. That prompted us to combine the two annoyance items, but the resulting scale was rather crude [52] because our study was not specifically designed to investigate noise perception.

Finally, data were collected in October and November, when people spend less time outdoors than in summer. That could have diminished the associations, at least for larger buffers. Therefore, we adjusted the models for month of data collection. Limitations notwithstanding, the associations we found were significant, lending support to our hypothesis.

## 5. Conclusions

Higher greenness and more green space in the residential environment were associated with lower noise annoyance, whereas for higher tree cover, this association was observed only in the 100 m buffer zone. Observed associations between greenspace and noise annoyance were mediated by higher perceived greenspace and lower noise level, and these paths seemed equally important. In the case of tree cover, only perceived greenspace was a mediator. Our findings suggest that the potential of greenspace to reduce noise annoyance extends beyond noise abatement. Applying a combination of GIS-derived and perceptual measures should enable researchers to better capture individual experiences of greenspace and to understand how it may relate to noise annoyance.

## Figures and Tables

**Table 1:** Pearson correlations of the main variables in the analysis (*N* = 720).

Variable	(1)	(2)	(3)	(4)	(5)	(6)	(7)	(8)	(9)	(10)	(11)	(12)	(13)	(14)	(15)
(1) Traffic NA	1	**0.47**	**0.85**	**0.17**	**−0.15**	−0.07	**−0.08**	**−0.11**	−0.04	0.01	0.04	−0.08	−0.07	**−0.10**	−0.07
(2) Neighborhood NA		1	**0.86**	**0.11**	**−0.18**	**−0.18**	**−0.18**	**−0.15**	**−0.10**	−0.03	0.03	**−0.10**	−0.07	−0.04	**−0.09**
(3) Total NA			1	**0.16**	**−0.19**	**−0.14**	**−0.15**	**−0.15**	**−0.08**	−0.01	0.04	**−0.10**	**−0.08**	**−0.08**	**−0.09**
(4) L_day_				1	**−0.15**	**−0.28**	**−0.26**	**−0.17**	−0.07	0.02	0.07	**−0.17**	**−0.25**	**−0.25**	**−0.15**
(5) Perceived GS					1	**0.29**	**0.24**	**0.20**	**0.13**	**0.09**	0.06	**0.14**	**0.12**	**0.08**	**0.10**
(6) NDVI _100m_						1	**0.75**	**0.53**	**0.47**	**0.17**	0.04	**0.21**	**0.34**	**0.26**	**0.12**
(7) NDVI _300m_							1	**0.83**	**0.25**	**0.14**	0.05	**0.20**	**0.58**	**0.49**	**0.13**
(8) NDVI _500m_								1	**0.15**	**0.18**	**0.16**	**0.22**	**0.42**	**0.48**	**0.23**
(9) Tree cover _100m_									1	**0.73**	**0.56**	**0.16**	−0.02	−0.04	**−0.12**
(10) Tree cover _300m_										1	**0.88**	**0.10**	**−0.16**	**−0.15**	**−0.19**
(11) Tree cover _500m_											1	0.04	**−0.14**	**−0.11**	**−0.27**
(12) GS% _100m_												1	**0.49**	**0.34**	0.05
(13) GS% _300m_													1	**0.86**	−0.03
(14) GS% _500m_														1	0.05
(15) Distance to GS															1
Mean	1.58	1.54	1.56	67.05	2.98	0.35	0.36	0.36	6.03	6.31	6.56	2.71	11.42	13.37	398.15
Standard deviation	1.09	1.13	0.95	1.73	1.10	0.08	0.06	0.05	6.66	4.44	3.48	8.66	12.86	12.16	1024.64

Notes: GS: greenspace, L_day_: day average noise level, NA: noise annoyance, NDVI: Sentinel-derived Normalized Difference Vegetation Index. Boldface indicates statistically significant coefficients (*p* < 0.05).

**Table 2:** Parallel mediation models linking Geographic Information System (GIS)-derived greenspace to noise annoyance (*N* = 720).

GIS-Metrics	Total Effect	Direct Effect	Indirect Paths (% of the Total Effect Explained)	
			L_day_	Perceived GS
NDVI_100m_	−0.16 (−0.26, −0.06) ^1^	−0.08 (−0.19, 0.02)	−0.04 (−0.07, −0.02) ^1^ (25%)	−0.04 (−0.08, −0.01) ^1^ (25%)
NDVI_300m_	−0.26 (−0.39, −0.13) ^1^	−0.18 (−0.31, −0.05) ^1^	−0.04 (−0.07, −0.01) ^1^ (15%)	−0.05 (−0.09, −0.02) ^1^ (20%)
NDVI_500m_	−0.16 (−0.25, −0.07) ^1^	−0.12 (−0.21, −0.03) ^1^	−0.01 (−0.03, −0.002)^1^ (6%)	−0.03 (−0.06, −0.01) ^1^ (19%)
Tree cover_100m_	−0.07 (−0.12, −0.01) ^1^	−0.05 (−0.10, 0.001)	−0.003 (−0.01, 0.004) (4%)	−0.01 (−0.03, −0.003) ^1^ (14%)
Tree cover_300m_	−0.05 (−0.14, 0.03)	−0.04 (−0.12, 0.05)	0.003 (−0.01, 0.02) (−6%)	−0.02 (−0.04, −0.01) ^1^ (40%)
Tree cover_500m_	−0.04 (−0.15, 0.07)	−0.02 (−0.13, 0.09)	0.01 (−0.003, 0.03) (−25%)	−0.03 (−0.06, −0.01) ^1^ (75%)
GS%_100m_	−0.05 (−0.09, −0.01) ^1^	−0.03 (−0.07, 0.01)	−0.01 (−0.03, −0.004) ^1^ (20%)	−0.01 (−0.02, −0.003) ^1^ (20%)
GS%_300m_	−0.04 (−0.07, −0.01) ^1^	−0.02 (−0.05, 0.01)	−0.01 (−0.02, −0.003) ^1^ (25%)	−0.01 (−0.01, −0.002) ^1^ (25%)
GS%_500m_	−0.04 (−0.07, −0.004) ^1^	−0.03 (−0.06, 0.01)	−0.01 (−0.01, −0.002) ^1^ (25%)	−0.004 (−0.01, <0.001) (10%)
Distance to GS	−0.01 (−0.03, 0.01)	−0.002 (−0.02, 0.02)	−0.01 (−0.02, 0.00) (100%)	−0.002 (−0.01, 0.001) (20%)

Notes: GS: greenspace, L_day_: day average noise level, NDVI: Sentinel-derived Normalized Difference Vegetation Index. Models adjusted for age, sex, ethnicity (Bulgarian vs. other), individual-level economic status, duration of residence (<5 vs. ≥5 years), time spent at home/day (<8 vs. ≥8 h), stressful life events (no vs. yes), population in 500 m buffer, settlement (Plovdiv vs. other), presence of bluespace in the respective buffer (Distance to green space is adjusted for distance to bluespace) and month of data collection (October vs. November); in addition, L_day_ and Perceived GS are mutually adjusted for each other. Coefficients for NDVI and Distance to green space are rescaled to an interquartile range increment, and for Tree cover and GS%, to a 5% increment. Effect size coefficient is unstandardized regression coefficient with its 95% confidence interval. ^1^ Coefficient is statistically significant at *p* < 0.05.

## Appendix A

**Table A1:** Parallel mediation models linking Geographic Information System (GIS)-derived greenspace to traffic- and neighborhood noise annoyances (*N* = 720).

GIS-Metrics	Total Effect	Direct Effect	Indirect Paths	
			L_day_	Perceived GS
Traffic Noise Annoyance				
NDVI _100m_	−0.11 (−0.22, 0.01)	0.002 (−0.12, 0.12)	−0.06 (−0.10, −0.03) ^1^	−0.05 (−0.09, −0.02) ^1^
NDVI _300m_	−0.18 (−0.33, −0.03) ^1^	−0.06 (−0.22, 0.09)	−0.05 (−0.10, −0.02) ^1^	−0.06 (−0.11, −0.02) ^1^
NDVI _500m_	−.13 (−0.23, −0.02) ^1^	−0.07 (−0.18, 0.03)	−0.02 (−0.05, −0.002) ^1^	−0.03 (−0.07, −0.01) ^1^
Tree cover _100m_	−0.04 (−0.10, 0.02)	−0.02 (−0.08, 0.04)	−0.005 (−0.02, 0.005)	−0.01 (−0.03, −0.005) ^1^
Tree cover _300m_	−0.01 (−0.11, 0.09)	0.01 (−0.08, 0.11)	0.004 (−0.01, 0.02)	−0.02 (−0.05, −0.01) ^1^
Tree cover _500m_	0.01 (−0.12, 0.14)	0.03 (−0.10, 0.16)	0.02 (−0.004, 0.05)	−0.04 (−0.07, −0.01) ^1^
GS% _100m_	−0.04 (−0.08, 0.01)	−0.01 (−0.06, 0.04)	−0.02 (−0.03, −0.01) ^1^	−0.01 (−0.02, −0.004) ^1^
GS% _300m_	−0.04 (−0.07, −0.003) ^1^	−0.02 (−0.06, 0.01)	−0.01 (−0.02, −0.004) ^1^	−0.01 (−0.02, −0.002) ^1^
GS% _500m_	−0.05 (−0.09, −0.02) ^1^	−0.04 (−0.08, −0.002) ^1^	−0.01 (−0.02, −0.003) ^1^	−0.005 (−0.01, <0.001)
Distance to GS	−0.01 (−0.03, 0.02)	0.003 (−0.02, 0.03)	−0.01 (−0.03, −0.0003) ^1^	−0.002 (−0.01, 0.002)
**Neighborhood Noise Annoyance**				
NDVI _100m_	−0.22 (−0.34, −0.10) ^1^	−0.17 (−0.30, −0.05) ^1^	−0.02 (−0.05, 0.01)	−0.03 (−0.07, −0.002) ^1^
NDVI _300m_	−0.35 (−0.50, −0.19) ^1^	−0.30 (−0.46, −0.14) ^1^	−0.02 (−0.05, 0.005)	−0.03 (−0.08, 0.004)
NDVI _500m_	−0.20 (−0.30, −0.09) ^1^	−0.17 (−0.28, −0.06) ^1^	−0.01 (−0.02, 0.001)	−0.02 (−0.05, −0.001) ^1^
Tree cover _100m_	−0.10 (−0.16, −0.03) ^1^	−0.08 (−0.15, −0.02) ^1^	−0.002 (−0.01, 0.001)	−0.01 (−0.03, −0.001) ^1^
Tree cover _300m_	−0.10 (−0.20, −0.001) ^1^	−0.08 (−0.18, 0.01)	0.002 (−0.004, 0.01)	−0.02 (−0.04, −0.003) ^1^
Tree cover _500m_	−0.09 (−0.23, 0.04)	−0.08 (−0.21, 0.06)	0.01 (−0.001, 0.03)	−0.03 (−0.06, −0.01) ^1^
GS% _100m_	−0.06 (−0.10, −0.01) ^1^	−0.04 (−0.09, 0.004)	−0.01 (−0.02, 0.0004)	−0.01 (−0.02, −0.002) ^1^
GS% _300m_	−0.03 (−0.07, 0.001) ^1^	−0.03 (−0.06, 0.01)	−0.004 (−0.01, 0.001)	−0.01 (−0.01, −0.001) ^1^
GS% _500–m_	−0.02 (−0.06, 0.02)	−0.01 (−0.05, 0.03)	−0.003 (−0.01, 0.0004)	−0.004 (−0.01, 0.0001)
Distance to urban GS	−0.01 (−0.04, 0.01)	−0.01 (−0.03, 0.02)	−0.003 (−0.01, 0.001)	−0.002 (−0.01, 0.001)

Notes: GS: greenspace, L_day_: day average noise level, NDVI: Sentinel-derived Normalized Difference Vegetation Index. Models adjusted for age, sex, ethnicity (Bulgarian vs. other), individual-level economic status, duration of residence (<5 vs. ≥5 years), time spent at home/day (<8 vs. ≥8 h), stressful life events (no vs. yes), population in 500 m buffer, settlement (Plovdiv vs. other), presence of bluespace in the respective buffer (Distance to green space is adjusted for distance to bluespace) and month of data collection (October vs. November); in addition, L_day_ and Perceived GS are mutually adjusted for each other. Coefficients for NDVI and Distance to green space are rescaled to an interquartile range increment, and for Tree cover and GS%, to a 5% increment. Effect size coefficient is unstandardized regression coefficient with its 95% confidence interval. ^1^ Coefficient is statistically significant at *p* < 0.05.
